# Materials Informatics for Mechanical Deformation: A Review of Applications and Challenges

**DOI:** 10.3390/ma14195764

**Published:** 2021-10-02

**Authors:** Karol Frydrych, Kamran Karimi, Michal Pecelerowicz, Rene Alvarez, Francesco Javier Dominguez-Gutiérrez, Fabrizio Rovaris, Stefanos Papanikolaou

**Affiliations:** 1 NOMATEN Centre of Excellence, National Centre for Nuclear Research, ul. A. Sołtana 7, 05-400 Swierk-Otwock, Poland; karol.frydrych@ncbj.gov.pl (K.F.); kamran.karimi@ncbj.gov.pl (K.K.); michal.pecelerowicz@ncbj.gov.pl (M.P.); Rene.Alvarez@ncbj.gov.pl (R.A.); javier.dominguez@ncbj.gov.pl (F.J.D.-G.); fabrizio.rovaris@ncbj.gov.pl (F.R.); 2 Institute for Advanced Computational Science, Stony Brook University, Stony Brook, NY 11749, USA

**Keywords:** metal alloys, machine learning, informatics, defects, dislocations, mechanical deformation, data science, ontology

## Abstract

In the design and development of novel materials that have excellent mechanical properties, classification and regression methods have been diversely used across mechanical deformation simulations or experiments. The use of materials informatics methods on large data that originate in experiments or/and multiscale modeling simulations may accelerate materials’ discovery or develop new understanding of materials’ behavior. In this fast-growing field, we focus on reviewing advances at the intersection of data science with mechanical deformation simulations and experiments, with a particular focus on studies of metals and alloys. We discuss examples of applications, as well as identify challenges and prospects.

## 1. Introduction

Materials informatics (MI) is an interdisciplinary field of study at the interface of material engineering and data science, which aims to boost discovery, characterization, component design, synthesis, and screening of new or alternative materials based on modern state-of-the-art machine learning (ML) techniques [1,2,3,4,5,6]. The Materials Research Society bulletin provides an exhaustive list across almost all aspects of materials science and engineering that are greatly influenced by this relatively new field, including (but not limited to) thermoelectric materials, superconductors, novel bio-materials, batteries, fuel cells, dielectric elastomers and so on [7,8,9]. An emerging category pertains to deformation and failure in metal alloys owing to an ever-increasing demand for materials that may withstand extreme conditions (temperature, pressure, loading rate), and also form optimal combinations of material weight, strength, ductility, corrosion-resistance, and toughness as essential infrastructural and industrial components [10]. In this framework, ML has been proven to be an efficient and powerful computational tool in micro-structural alloy tailoring and characterization [11,12,13,14], with robust predictions of fundamental thermomechanical and physical properties [15,16,17,18,19,20,21,22], and systematic development of improved atomic potentials based on high-throughput material computations [23,24,25,26].

Materials data scientists make routine use of advanced statistical learning elements [27] (i.e., regression, classification, regularization, dimensionality reduction, and cross-validation [28]) for insights into inherent patterns, underlying physics, and structure-property correlations across a broad range of length-scales and time-scales [25]. It is indeed a fact that only very few of these features can be systematically explored solely based on theory, experiments, or computations, and thus, prediction of complex materials behavior has been limited. The main challenge concerns the high-dimensional and scattered nature of the parameter space associated with multi-component materials, making the design problem intractable based on conventional materials science approaches [29]. High entropy alloys, for instance, are formed by mixing multiple principal components with a tendency to form unary, binary, or more complex phases that may also undergo phase transformations depending on additional processing [30]. A massive combinatorial phase space exists, even though it has only been partially investigated through experiments and/or simulations. ML provides possible frameworks to integrate search strategies for novel materials with tailored properties in a timely, yet robust and efficient manner, which is otherwise impossible using traditional frameworks.

**Figure 1:**
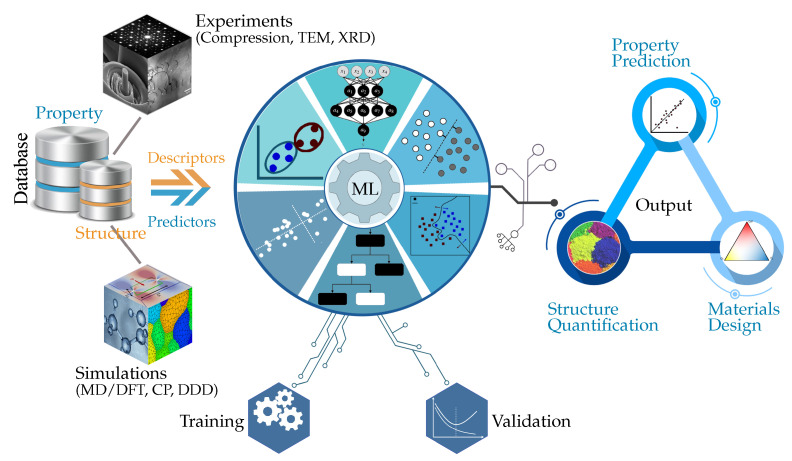
Typical materials informatics and machine learning workflow for mechanical deformation. Databases (**left**) generated through microscopy and (nano)mechanics experiments are combined with multiscale modeling simulations to provide relevant descriptors and predictors. The latter are used to train and validate ML algorithms (**center**) leading to the property prediction, structural characterization, and materials design/discovery (**right**).

As schematically illustrated in Figure 1, the implementation of an ML-based approach involves two elementary steps: pattern detection of input experimental or/and simulation data, and predictions, both essential in understanding fundamental material properties [31]. The former demands continuous access to sufficient amounts of data which are essential for any quantitative assessment of materials behavior. The sources of data are typically diverse, including those (re)produced from high throughput (ab initio, atomistic, and continuum-based) simulations, and extensive experimentation (typically, mechanical testing and microscopy) and/or those collected from existing materials databases [32,33,34]. In the informatics study of structure-property relationships, a relevant material dataset contains a feature space normally spanned by a set of atomic and/or local (micro-)structural descriptors that presumably capture salient physical mechanisms and microstructural details for an existing set of materials [17]. This information may cover a broad range of attributes including element-wise atomic properties [15,35,36,37] and atomic fractions in multicomponent materials [16,38,39]. But, also, it may involve topological measures, such as coordination number, packing efficiency, and local ordering [17,40], lattice distortion [15,41], and local crystallography [42] in metallic systems.

**Figure 2:**
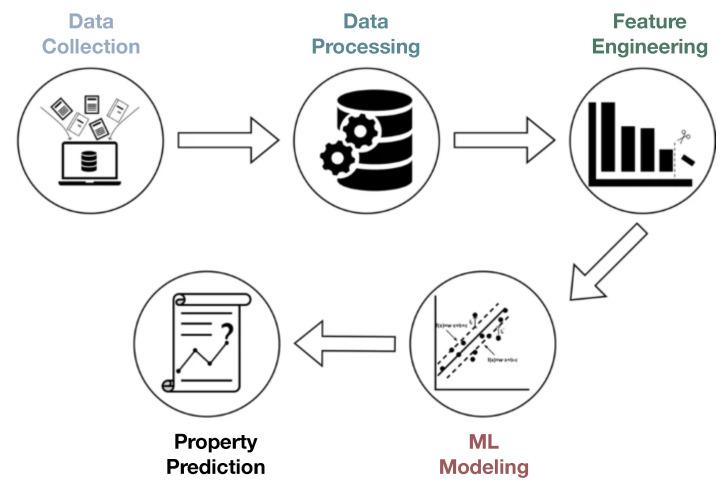
Main steps towards property predictions through the use of ML frameworks, driven by feature engineering in materials science. For mechanical deformation, multiscale microscopy and testing experimental data, as well as multiscale modeling data are used as input, that may exceed 1PB. From there, a key step involves data processing and reduction approaches [45,46], that may allow the derivation of meaningful features that capture key physical mechanisms. As soon as features are consistently defined, property prediction and machine learning models are built. Currently, the biggest challenge is focused on feature engineering and data management. Redrawn based on Ref. [47].

In an unsupervised learning context, ML users typically seek to analyze underlying trends, clustering features, and multivariate correlations associated with the input features which are presumably tied to distinct physical processes governing certain (bulk) material properties. In contrast, supervised ML methods additionally incorporate a set of predictors, commonly attributed to fundamental material constants or properties, which are potentially influenced by descriptors but without any a priori knowledge about the degree of inter-correlations. It should be noted that, depending on the context, a meaningful combination of local and global attributes may serve as features datasets in order to make predictions and optimizations on a different set of bulk physical properties as target data (cf. Figure 2). For metallic alloys, for instance, Vickers hardness may relate to intrinsic inter-atomic interactions, but also strongly correlates with elasticity, yielding, and toughness [43,44]. The predictive aspect of ML assists in establishing a quantitative structure-property association with no prior assumptions and purely based on existing materials data, playing a key role in the study of novel engineering materials.

There are several reviews in the scientific literature with a theme focused on applications of data analytics in materials science [29,31], data analytics and image processing for microstructural identification and physics inference [5] as well as data-centric science relevant to thermodynamics/mechanics in different families of alloyed metals (including metallic glasses and high entropy alloys) [14] and other structural (continuum-based) materials [48,49] or bulk glassy systems [50], and learning methods on searching for property-related descriptors [29,51]. The focus of our work is to concentrate on MI applications that are dedicated to the modeling and prediction of mechanical properties, such as elastic moduli, yield points, hardness, etc. The importance of this endeavor is to incorporate the complexity of physical mechanisms associated with mechanical deformation into the conversation of MI applicability and transferability. The multiscale character of mechanical defects in crystalline and amorphous metals requires the efficient use of multiscale modeling approaches in tandem with the application of ML methods. In this fast-growing field, our review aims only to capture the common aspects of utilized MI methods and frameworks, and identify the key challenges that have recently emerged.

In this context, a major success of MI methods is typically identified by novel material discovery, as an essence of the inverse design framework which aims to connect properties to underlying mechanisms related to deformation and failure in alloyed metals [52]. A special focus will be made on the reference to such works, and also on the extraction of relevant physics from simulation-based and experimentally-derived (image) data. Numerical simulation frameworks focus on mechanical deformations at the microstructural level, and they range from first-principles ab initio calculations and atomistic (Molecular Dynamics and Monte Carlo) simulations to coarse-grained mesoscale frameworks (Discrete Dislocation Dynamics) and phenomenological continuum models, all well-established techniques within the computational materials science community. The experiment-based contributions will be mainly focused on mechanical testing data, as well as microscopy defect feature identification from high-resolution electron microscopy, X-ray scattering, and digital image correlation (e.g., irradiation defects, dislocations in crystals, grain boundaries in polycrystals, quenched disorder in metallic glasses, or micro-cracking in brittle solids). It is worth noting that, in the context of mechanical deformation, the quantification of various defect types can be enough information to safely predict mechanical responses in various experimental set-ups. Further discussions will follow on how the outcomes of such an analysis may improve existing lattice-based and continuum models by incorporating key micro-mechanical/structural ingredients (e.g., defect density, dislocation interactions).

The paper is organized in a way that elucidates MI applications for the prediction and inference of mechanical properties and novel material designs, starting from self-evident features and ending with intricate engineering of nanoscale inverse alloy designs. Section 2 begins with a focus on image-based characterization techniques combined with advanced machine learning tools to explore relevant microstructural features in metals and alloys, that are typically used as descriptors for modeling and prediction of mechanical properties in metals and alloys. We highlight, in particular, applications of deep learning in materials classification, phase identification, topological characterization, and physics extraction in both supervised and unsupervised contexts. In Section 3, we turn to mechanical deformation experiments and highlight the machine learning capabilities in identifying prominent features that are intrinsically linked to desired physical and mechanical properties. Focusing on crystalline alloys, in Section 4, we move to a discussion of the classifications of dislocation ensembles and crystal plasticity, on the basis of combined discrete dislocation dynamics and machine learning frameworks. The classification of dislocation ensembles represents a promising direction for understanding microstructural processing routes. We further present several ML-based illustrations of property derivation from a set of high-resolution strain images that contain important information about dynamical mechanisms of dislocation ensembles. Validation of dislocation-related mechanisms may only be performed at the nanoscale, so Section 5 considers the application of materials data science in the in situ characterization and classification of nanomechanics experiments. Section 6 explores the current state-of-the-art in using machine learning and mechanical properties related to materials discovery and high-throughput metal-alloy design. Finally, given that data management and transferability issues represent key bottlenecks in MI applications, Section 7 provides a brief, but highly important, discussion on the distinct aspects of materials information ontology relevant to the data-driven research of materials deformation as well as a broad overview of associated impacts, existing challenges, and prospects.

## 2. Materials Informatics in Microstructural Image Classification

A common approach towards MI applications has been the use of large imaging datasets to obtain defect densities that are linked to mechanical properties of given materials. Modern data science and ML methods enable automated analysis of data coming from various imaging techniques, often in combined forms. Images can be prepared using various experimental techniques, and in the context of mechanical deformation, they include optical microscopy (OM), digital image correlation (DIC and HR-DIC) for the analysis of strain information, scanning electron microscopy (SEM), transmission electron microscopy (TEM) and electron back-scatter diffraction (EBSD). Characteristic applications, in this context, include the automatic identification of key defected regions that may compromise mechanical properties, and examples of challenging, but exemplary applications, such as the classification of irradiation-induced defects.

Early applications of informatics methods on microstructure imaging emerged in the context of OM. Using random forest (RF) algorithms [53,54] and unsupervised learning approaches [55], the steel micro-constituent segmentation based on OM images was achieved. Beyond OM, and in the context of mechanical properties, DIC has emerged as a promising technique. DIC uses specimen surface images taken in the initial and deformed configuration to produce displacement and strain fields. Although DIC is a computationally-intensive technique [56], some recent progress suggests that its speed can be increased by the application of convolutional neural networks (CNNs) [57], and therefore the potential for big data sets may fully develop. Recently, it has been noticed [58,59] that DIC results contain very rich information related to plastic deformation gradients, but only a part of it is utilized. Through the use of unsupervised ML approaches [58,60] it is possible to gain insights into the history of deformation of the crystalline sample, as well as consistently predict mechanical properties such as the yield stress, without the use of stress information. Furthermore, ML methods have been applied to determine the fracture toughness of composite materials based on DIC results, typically using artificial neural networks (ANNs) [45,61,62]. Crack detection, measurement, and characterization based on DIC can be performed using image processing methods [63] and fatigue crack detection in DIC images may be automatically performed using CNNs [64].

**Figure 3:**
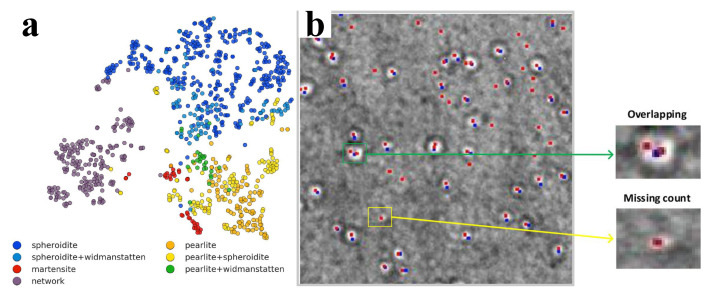
Microscopy and machine learning for identifying defects strongly related to mechanical properties. (**a**) The map of t-distributed stochastic neighborhood embedding (t-SNE) for the entire ultrahigh carbon steel dataset. Spheroidite, pearlite, etc. are various steel microstructure constituents, the densities of which define steel mechanical properties and are commonly classified by human experts. Here, an automated approach can provide steels’ mechanical property estimates. The figure is reproduced after [11] with permissions from Elsevier. (**b**) The location of He bubbles in a TEM image of an irradiated Ni-based alloy marked manually by TEM analysis experts (blue) and using the ML approach (red). Reproduced after [68] based on Creative Commons CC BY license.

At the nanoscale, transmission electron microscopy (TEM), SEM, and EBSD can be used to prepare image libraries for training ML frameworks. An important application of ML trained on SEM images is the steel microstructure classification. In Refs. [11,12], CNNs were applied to classify microconstituents of ultrahigh carbon steels (UHCS). This is a characteristic application that can be extended in a large collection of material classes [45]. Figure 3a presents the t-distributed stochastic neighborhood embedding (t-SNE) map for the UHCS data set and the constituents were classified based on SEM images. In Refs. [65,66] the fully convolutional neural network (FCNN) accompanied by a max-voting scheme was applied to classify microconstituents of steel. For this goal, fine-tuning and data augmentation were applied. The ML framework was trained on both SEM and light OM images. Beyond neural networks [67], the classification of steel microstructures can be performed by the application of support vector machines (SVM), or RF assisted with gradient boosting machines.

It is possible to combine DIC with an SEM image collection to perform high-resolution digital image correlation (HR-DIC). In this way, it is not only possible to classify material microstructures, but also to investigate novel deformation mechanisms. For example, the identification of deformation twins in HR-DIC of Mg alloys was undertaken in [69,70].

The use of SEM technology has also led to the emergence of the EBSD technique as an important data-rich experimental method commonly used to investigate the microstructure of polycrystalline materials. An application of ML methods in this context is the segmentation and classification of patterns such as grains and grain boundaries. Segmentation methods may directly incorporate the detection of anomalies and ML methods can be applied to increase the speed of indexing and classification of EBSD patterns as in [71,72,73,74]. Given that EBSD connects to lattice misorientations in crystals, another application of MI methods includes physics extraction based on the acquired EBSD data. For example, ML methods can be used [75] to find correlations between microstructural parameters and twinning, by using decision trees, ultimately identifying the influence of various crystallographic and morphological attributes on twin nucleation and propagation in AZ31 Mg alloys. By connecting to dislocation physics [76], it is also possible to apply deep learning to estimate the total dislocation density based on a geometrically necessary dislocation (GND) density. The method was applied to EBSD images of α-Fe.

At the atomic scale, TEM and high-resolution transmission electron microscopy (HRTEM) are efficient experimental techniques revealing detailed microstructural information. However, as the analysis of TEM and HRTEM images is a highly tedious and labor-consuming task, it is an open challenge to perform materials informatics to automate TEM image classification. Nevertheless, in [77], deep convolutional neural networks trained on simulated data were only able to interpret HRTEM images and the analysis of nanoparticles observed in TEM [78] based on an evolutionary algorithm (EA) may be applied, while the performance of various CNNs for the task of TEM image segmentation has been evaluated in [79]. However, an important problem is the point defect detection in TEM images. To resolve this challenge, a possible approach is to use supervised ML that requires simulation-generated data with appropriate labels [77,80]. It is also possible to consider, as in [81], the problem of defect detection as an anomaly detection one: in such a case, self-supervised ML could be only trained on defect-free experimental images. When there is no imaging noise, applying principal component analysis (PCA) can be shown to be enough to locate defects, but CNNs may improve the model performance in the presence of imaging noise that may also originate in quenched disorder [45,52,58,59,82]. Another way to circumvent the noise issues in TEM is by using scanning transmission electron microscopy (STEM) [80] where the size and quality of STEM datasets have been increasing exponentially. For example, artificial neural networks were applied [83] towards the detection of twins in STEM images [84,85].

A characteristic challenge in defect classification for mechanical properties is irradiation. In contemporary and future nuclear fission and fusion power plants, irradiation inherently leads to changes of the material microstructure [86], in ways that are highly complex in terms of predictive abilities and combination of processing routes, posing an immense challenge in the inference of irradiated structure-property relationships. In particular, the nature and variability of defects across scales, irradiation dose, and temperature, as well as the nature of the irradiation beam of incoming particles (alpha, beta, ions) is highly complicated. Despite the apparent complexity, MI methods can facilitate defect classification. A characteristic example was shown through the use of STEM images of irradiated steels that were analyzed using ML approaches [87], in particular CNNs, leading to the identification of irradiation-induced defects such as dislocation loops. Furthermore, it has been possible to apply ML to detect He bubbles in TEM images of Ni-based alloys irradiated with He ions at 650 ${}^{\circ }$C. The comparison of manually and automatically detected He bubbles is shown in Figure 3b. Finally, an automated system for analyzing in situ TEM videos was presented in Ref. [88]. where a deep-learning tool called YOLO (you only look once) can be used to analyze dislocation loops present in FeCrAl alloys irradiated to various damage doses.

## 3. Informatics in Deformation Experiments and Simulations

While detecting and classifying defects can lead to insightful conclusions, their behavior in situ as the sample is mechanically loaded, provides a unique view in the structure-property relationships that become accessible through materials informatics. Indeed, for material design, a combination of optimal ductility and strength requires prior knowledge about the nucleation and growth of relevant microstructural phases as well as their consequences in terms of macroscopic properties of interest, a training task that seems quite amenable to ML.

### 3.1. Searching for Microstructural Features and Machine Learning

**Figure 4:**
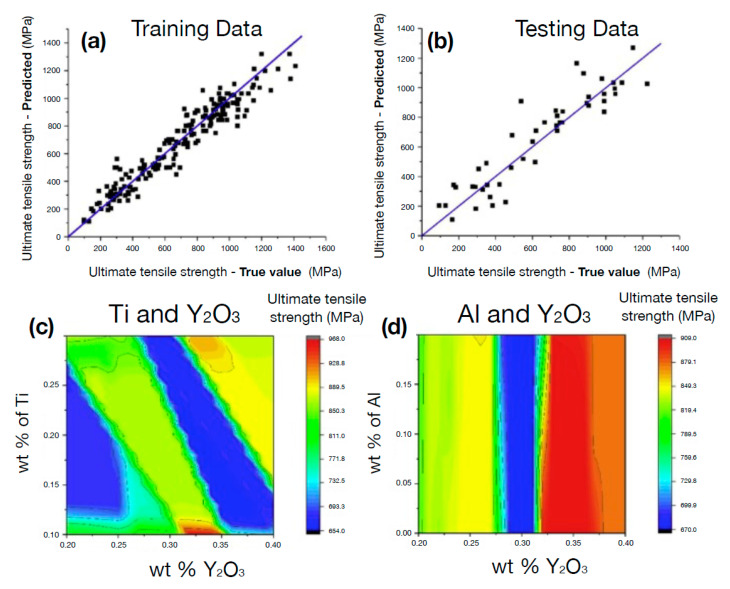
Integrated materials informatics to discover high-strength ductile alloys. A yield strength model was utilized quantitatively to identify contributions of solid-solution strengthening and grain refinement hardening into composition-structure-property relationships. (**a**,**b**) Model validation on the prediction of yield strength, (**c**,**d**) prediction of composition-dependent strength. Adopted from Ref. [89] with permissions from Elsevier.

Bulk elasticity of metals, as a main functional property, is largely controlled by electronic structures, chemical bonding, atomic/molecular arrangements, preparation history, and thermal treatments, forming an intractable design space from a materials engineering point of view. The materials science literature provides numerous instances where an appropriate combination of surrogate data-driven models and deformation-based experiments led to robust and meaningful predictions of key functional properties out of a tremendous feature space (for example, see Figure 4 for a characteristic application for the prediction of ductile, high-strength alloys [89]). Earlier attempts in this context originated in the use of data analytics (neural networks in particular) in flow stress prediction of metallic materials [90], aluminum alloys [91,92], steel [93,94], Ti alloy [95] under varying temperature, deformation rates, and applied strains. Neural network training was also practiced in terms of the alloy composition search that, along with heat treatment parameters, lead to accurate evaluations of yield strength, hardness, ductility, elasticity, fatigue properties, and fracture toughness [96,97,98,99,100,101,102]. Furthermore, in [15], an extensive neutron-diffraction characterization was carried out to obtain elastic constants associated with an Al${}_{0.3}$ CoCrFeNi alloy under uniaxial tension. The empirical measurements along with an appropriate set of relevant elemental parameters (i.e., group number, cohesive energy, density, atomic radii) were used to feed a gradient-boosted trees algorithm. More generally, intensive research effort was undertaken in the field of amorphous metals. For example in Ref. [37], a set of dynamically-measured elastic moduli based on ultrasonic-wave-propagation tests led to an SVM regressor that was used to relate bulk and shear moduli to a set of training features, with the atomic electronegativity, atomic volume, and atomic size differences being selected as best-performance descriptors.

**Figure 5:**
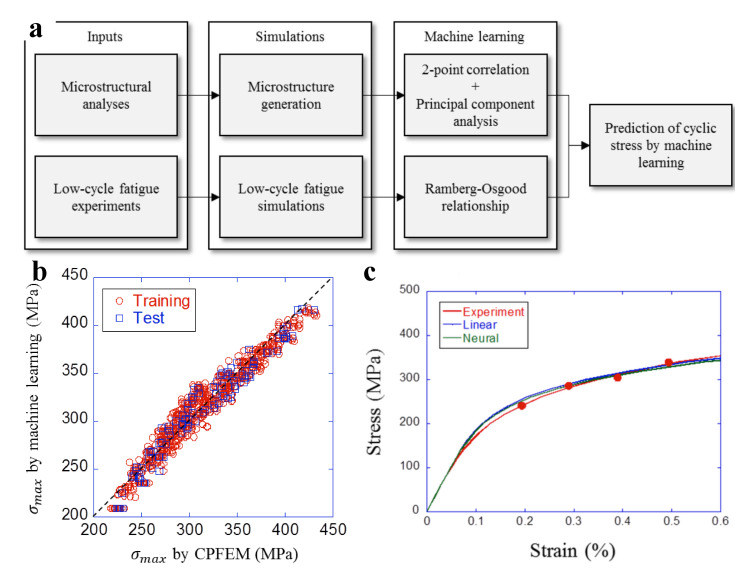
Machine learning to predict stress-strain curves in steel. (**a**) Framework applied by Miyazawa et al. [114] in order to predict the cyclic stress-strain property of dual-phase steels. The approach combines EBSD, mechanical experiments, and FEM simulations of crystal plasticity, to develop a principal component analysis ML model. (**b**) Prediction result of a linear regression model with the best combination of principal components. (**c**) Comparison results between machine learning and experiment for S25C dual-phase steel. Reproduced after [114] based on Creative Commons Attribution License.

However, beyond elasticity, the ability to predict mechanical properties is limited by the complexity of plastic deformation mechanisms. It has been clear for some time that, for realistic cases of structural materials (say, steels) and loading approaches (say, fatigue), MI success requires an intense interplay of fine-tuned MI frameworks that combine physical expectations with agnostic features’ definitions (see, for example, the application in Figure 5). Given that there are no standards in assessing MI applicability, it is also clear that the key sign of whether MI works for plasticity, has come from the MI investigation of the most complex applications in plasticity, namely amorphous metals. In the heavily-studied amorphous alloys, it has been known for many years now that local topological structures must have strong bearings on the physics and mechanics. There has been a growing body of research attempting to establish robust microstructural origins associated with common glassy features including slow relaxation dynamics, aging phenomenon, and dynamic heterogeneity (see [103] and references therein). The main focus has been on the prevalence of certain kinds of local ordering, purely based on (static) structural information, that will strongly correlate with dynamical properties. Commonly used structural quantities, representing potential ML features, include (but are not limited to) the coordination number, local potential energy, free volume [104], radial distribution function [40], close packing and local polyhedral order [104,105], or order parameters describing bond-orientational order in condensed phases [106]. It has been further argued that the predictive capacity, i.e., degree of correlations with dynamical quantities, associated with each individual structural descriptor (or in combination together) could significantly vary across a wide range of supercooled liquids and glasses [103,107]. Similar connections have also been established in the context of mechanically-driven glasses, where the local structure is suggested to provide strong evidence for bulk mechanical properties [108]. In metallic glasses, for instance, local atomic configurations are believed to strongly correlate with elastic heterogeneities [109,110]. Atomistic simulations of [111,112] revealed the emergence of local structural motifs that imprint soft environments susceptible to irreversible shear-induced rearrangements and, therefore, improve the plasticity of metallic glasses. This has been contrasted with “short-range ordering” in an amorphous matrix with insignificant contributions to the deformation mechanism but instead playing an important role in slowing down the dynamics of supercooled liquids [108]. In [113], an abundance of the former structural patterns was linked to the enhanced deformability in rejuvenated glass structures which is at odds with aging samples, generally rich in terms of the latter local features.

**Figure 6:**
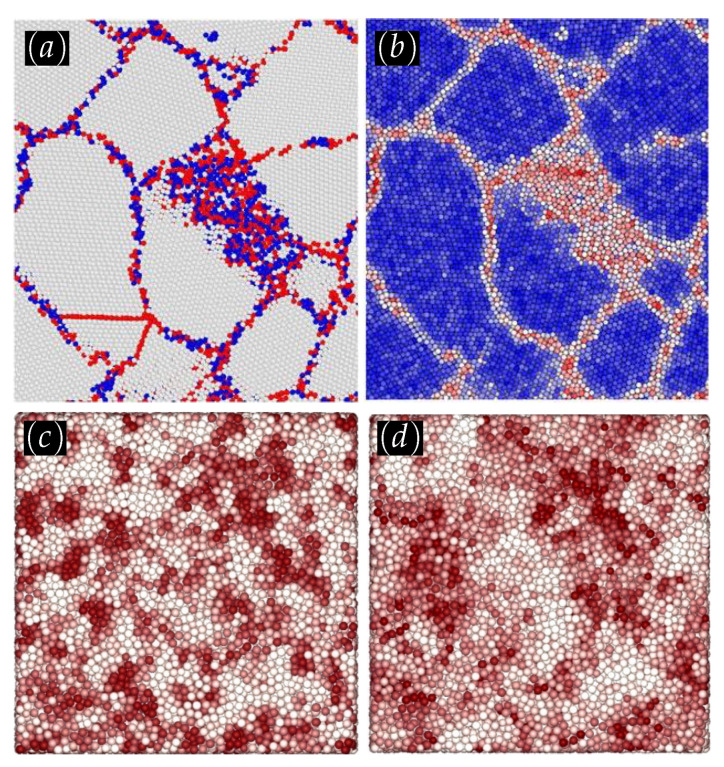
Linking microstructure with dynamics. (**a**) Distinct microstructures (bulk grains versus grain boundaries) illustrated based on the Voronoi topology analysis of a simulated polycrystalline aluminum. The white, red, and blue colors denote the FCC phase, hexagonal close-packed (HCP) structure, and atoms with neither the former nor the latter structure, respectively. (**b**) The same snapshot displaying the associated softness (reproduced from [42]). Atoms with a high softness are red and those with a low softness are blue. (**c**) Binary microstructural classification of particles in a model glass former colored according to the probability of being a member of the corresponding cluster. Dark red and white denote high and low probabilities. (**d**) The same particles but color-coded in accordance with the associated mobility. Dark red and white indicate high and low mobility (reproduced from [106] licensed under CC BY 4.0).

Pattern detection in the research of glassy matter is generally regarded as a supervised learning process where appropriate machine learning features (and/or regressors) are trained based on a large set of atom-wise dynamical measures (atomic trajectories, mobility, or rearrangements) and corresponding local structural information (e.g., average pair correlation function, coordination number, bond orientation etc.) [17,115,116,117]. The concept was largely put forward in a series of important papers applying machine learning to describe the interplay between structure and dynamics in several glass formers as well as polycrystals [42]. The former includes supercooled liquids and glassy solids [104,107,118,119] and sheared amorphous solids [104,120,121,122]. In this framework, machine learning models successfully learned from training datasets to distinguish a population of rearranging atoms from that of local frozen regions and predict mechanical properties. The developed methodology was further validated by its predictive capabilities in terms of the subsequent atomic mobility solely based on the existing local topology. The observed binary separation led to defining a local metric called “softness” by measuring a (signed) distance of each input feature from the predicted decision boundary within the multi-dimensional feature space. Physically speaking, the softness field should entail the atoms’ propensity to undergo localized rearrangements (as a dynamical quantity) based upon a fully structural notion (see Figure 6a,b). The authors further hypothesized and validated (numerically) that the softness probability has an Arrhenius-type dependence on the temperature. They were able to measure local energy barrier height accordingly. In the context of metallic glasses [40], a local dynamical metric called “flexibility volume” was defined based on the product of the vibrational mean squared displacement and average interatomic distance which is similar in essence to the notion of softness. Regression analysis led to a robust prediction of the structural flexibility based on local atomic environments and was shown to strongly correlate with a broad range of elasto-plastic properties. In the context of soft amorphous matter, rearrangements often take localized forms, such as T${}_{1}$-type transformations in foam dynamics, which may also be predicted through structural features characterizing the liquid-air interface [123]. Finally, several unsupervised learning frameworks have been developed with the goal being to describe the associations and patterns among a set of local structural measures [104,106] which further investigated strong correlations between unsupervised clustering and dynamics in classical model glass formers, as displayed in Figure 6c,d.

### 3.2. Materials Informatics and In Situ Loading: DIC and Surrogate Models Based on Plasticity

**Figure 7:**
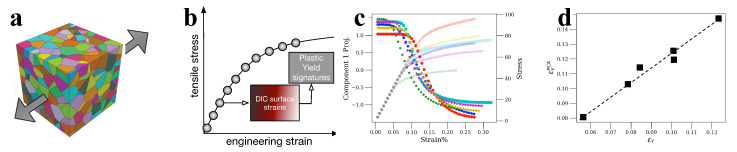
DIC and materials informatics for yield strength prediction in polycrystalline metals. (**a**) DIC on the top surface of a polycrystalline metal, studied through crystal plasticity simulations. (**b**) Strain map information acquired in situ can be processed to uncover yielding information, not accessible through common measures, (**c**) Unsupervised machine learning (PCA), in Ref. [60], a transition signature was identified in component projections, (**d**) yield point prediction through the proposed method, provides a very accurate alternative to common engineering ways by just studying stress-strain curves. Adopted from Ref. [60] with permissions from American Physical Society.

In the context of crystalline deformation and beyond elasticity in polycrystalline metals and alloys, the primary focus has been the use of DIC methods and then, the associated pursuit of plasticity models that reproduce strain behaviors. DIC methods produce huge amounts of strain information that typically concentrate into 1-2 colorful images in research publications, but with the concrete understanding that a wealth of properties may be accessible through MI methods. Characteristically, in this context, recent emulations of DIC data through polycrystalline pure Al simulations [60], showed that DIC data can be used, through the use of PCA, to derive sample yield strength, a property that is commonly thought to not be accessible by the sole information of total strains (see also Figure 7). In this way, it is clear that DIC has yet to be explored as a technique. However, MI applications, DIC-related, flourish, in a way that multiscale materials simulation methods are utilized to assist DIC features’ interpretations. On the microstructural level, several research papers have been devoted to coupling HR-DIC with plasticity: in [124], various materials (BCC tantalum, FCC Nickel, Ti-6Al-4V) have been investigated using a combination of crystal plasticity and finite element methods (CPFEM) and three experimental techniques, namely EBSD, HR-DIC and surface profilometry. The strain fields predicted by CPFEM were directly compared with HR-DIC measurements and very good agreements were obtained [125,126,127]. Another possibility along the same lines would be the identification of local elastic strains and lattice rotations by applying HR-EBSD. CPFEM and HR-EBSD were applied to investigate the heterogeneous elastic strains near the impurity-originated inclusion in nickel superalloy in [128,129].

In this context, an interesting materials informatics application was presented in [130,131]. The approach is based on coupling experimental techniques, crystal plasticity (CP) simulations and Bayesian networks to investigate the short crack growth in beta-metastable BCC titanium alloy (VST-55531) subjected to high cycle fatigue. The applied experimental technique was a combination of diffraction contrast tomography, multiple phase-contrast tomography scans, and modern reconstruction and segmentation techniques. Tree augmented Naive Bayes methods were applied to extract correlations between postulated short crack driving force metrics and experimental results. Another interesting application is the investigation of the cyclic stress-strain curves of various dual-phase steels differing in pearlite-to-ferrite proportion as predicted by Miyazawa et al. [114] (see also Figure 5). First, the microstructural analyses were conducted using EBSD in order to extract the grain morphology and determine the location of both phases. Then, the stress-strain curves obtained from low-cycle fatigue experiments were utilized to obtain the parameters of the macroscopic J2 plasticity model of pearlite phase and crystal plasticity model of ferrite. Then, 2D FEM simulations were conducted in order to provide the dataset for ML. Two ML frameworks, namely the linear regression and neural network, were applied to dimensionally reduced datasets thus enabling to provide cyclic stress-strain predictions.

Artificial neural networks (ANN) have been also shown to form useful MI applications. For example, in [132], an ANN was trained using data from the rate-dependent CPFEM modeling in order to predict mechanical response and texture evolution. The approach was applied to AA6063-T6 alloy to study the tension and simple shear of both single and polycrystals. The 2D CPFEM model was built based on EBSD results. The feed-forward backpropagation ANN was used. The strain and crystallographic orientation have been applied as an input vector. The output consisted of stress and updated orientation. Interestingly, it was demonstrated that the trained ML lead to correct predictions outside the bounding box as well. First, it predicted the correct stress-strain curves and the texture evolution at larger strain under simple shear. Second, it correctly predicted the stress-strain curves after changing the strain path. Note that, as the CPFEM results were demonstrated to be in good agreement with the experimental results, the ANN approach automatically provides predictions consistent with experimental data.

A key direction, in the domain of building a reduced-order model is the application of a materials knowledge systems (MKS) approach. The aim of this formulation as developed by Kalidindi and coworkers [133,134,135] was to optimize the microstructure of the material to meet the required properties. In [136] the MKS framework was extended to polycrystalline microstructures. Namely, CPFEM simulations for various artificial microstructures of α-Ti were performed. The MKS was then calibrated so that the elastic stiffness and yield strength under uniaxial tension obtained with PCA as compared to CPFEM results would provide the desired accuracy. This way, it is possible to obtain these two mechanical properties for a given texture much faster as compared to the CPFEM approach. Note, however, that the new texture should lie between the bounds of the calibration set and that the calibration would have to be repeated if one is interested in different boundary conditions or different mechanical properties. A similar approach was then applied to evaluate the fatigue performance first in the high cycle fatigue [137,138] and then in the transition fatigue regime [139].

## 4. Learning from Crystal Defects: Dislocation Ensembles

Dislocations and their dynamics have been a key tool towards understanding polycrystalline deformation in metals and alloys, so it is natural to pursue MI applications in this context. Discrete Dislocation Dynamics (DDD) has been one of the major tools used for the numerical simulation of mechanical properties of materials [140]. Dislocations are defects at the atomic scale, but their collective movement and interaction determine the mechanical properties of a material at the macroscopic scale. DDD relies on a spatial discretization of the dislocation lines, generally achieved by considering small straight segments [141] whose motion is dictated by the local stress field acting at their position through the Peach and Koehler expression [142]. The application of DDD approaches in the past three decades has provided major insights into the deformation behavior of materials [82,143,144], elucidating fundamental mechanisms underlying material deformation: size effects in plasticity [145,146,147,148,149,150,151], the role of dislocation junctions in material hardening [152,153,154,155] and the identification of dislocation avalanches [82,156,157,158,159,160,161] and associated events [123].

**Figure 8:**
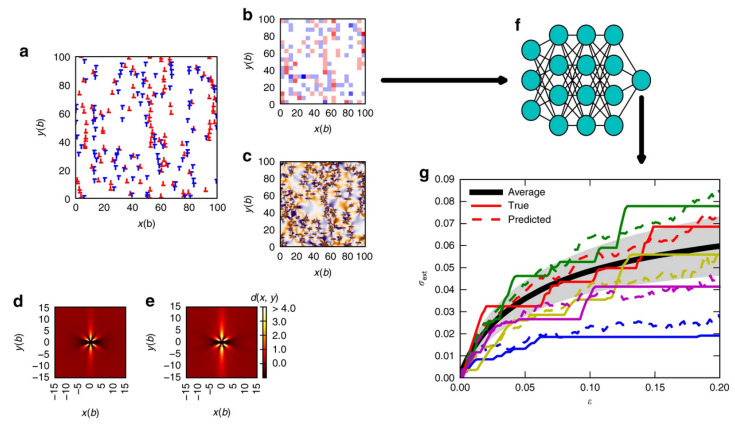
Workflow showing an ML approach for the prediction of mechanical properties of materials, based on DDD simulation data. Features are selected from the initial dislocation microstructure (**a**) like the geometrically necessary dislocations (GND) density of positive and negative dislocations (**b**) and the internal stress field (**c**). After defining, as initial states, relaxed random configurations (pair correlation function shown in (**d**)), or pre-deformed dislocation configurations (**e**), the authors trained a neural network (NN) (**f**) in the prediction of stress/strain curves based on these descriptors. Results showing NN predictions against true stress/strain curves are shown in (**g**). The figure was reproduced after [167] based on the Creative Commons Attribution 4.0 license.

Despite the wide range of successful applications achieved by DDD in the field of mechanical properties of materials, the advent of MI sets a new paradigm on how traditional DDD frameworks can be utilized towards insightful predictions for structure-property relationships [162,163,164,165,166]. The ability of ML models such as ANN to learn complex non-linear behaviors provides an opportunity to guide the understanding of complex collective dislocation behaviors and their influence on mechanical deformation. In this way, DDD can be regarded as a high-throughput approach to generate large data, to be analyzed by ML approaches in the search of new physical correlations, generally hidden by the ensemble and relevant mechanisms’ complexity. In this context, Ref. [167] demonstrated that it is possible in simple models to predict stress/strain curves as simulated by two-dimensional DDD simulations starting from features extracted from initially random dislocation microstructures. The importance of the result is encapsulated in the complexity of the initially random configurations, which may also acquire fractal characters [82,168,169]. The descriptors exploited in this work were features extracted from the initial dislocation microstructures (Figure 8a) such as the density of GNDs and the internal stresses as shown in Figure 8b,c, respectively. The authors addressed the issue of deformation predictability starting from systems with different sizes and also considering the effect of pre-straining the system before training (Figure 8d,e). Results regarding the performances of their NN-based model (Figure 8f) on the prediction of the stress-strain curves showed that better performances can be obtained for larger systems and higher deformation strain. Likewise, initial pre-deformation improves the predictive ability of the model. Similar topological descriptors were employed [170] to characterize precipitates-mediated jamming-to-pinning transition in terms of the dislocation network topology. Using dislocation structures as input, a confusion algorithm was successfully trained based on the binary classification of states according to the probability of being a member of the pinned or jammed phases. In more physically consistent conditions, Ref. [171] discussed high-throughput DDD simulations [58] towards generating synthetic experiments for 2D thin aluminum films, where the pre-processing route, in the context of the level of tensile pre-straining, controlled the initial dislocation configuration. Then, prediction of stress-strain curves and mechanical properties was achieved by repeating synthetic DIC experiments, and by using both unsupervised and deep-learning ANNs. The comparison between deep-learning and unsupervised learning methods showed a drastic advantage towards deep ANN approaches. In this way, progress in DIC techniques may provide a tool for a complete, non-destructive characterization of the mechanical behavior of materials.

Finally, it is worth noticing that MI methods may be used towards a complete classification of dislocation ensembles. While physical tools such as local misorientations, or topological features [45] may allow for ensemble classification, Ref. [172] investigated the possibility of defining ML descriptors for the classification of dislocation microstructures. The authors carried out DDD simulations with free surfaces in order to test different density fields commonly used in continuum models of dislocations such as the dislocation density, Nye’s tensor, and higher-order fields. In this context, it was possible to classify the sets of descriptors commonly used in Continuum Dislocation Dynamics (CDD). This result is important as it paves the way for a multi-scale framework where data are automatically passed from lower to higher-scale models. Other works [173] focused on calibrating CP models based on results from DDD by exploiting concepts from ML such as regularized regression and cross-validation.

Due to the inherent length-scale, DDD simulations have been considered as an ideal candidate for the direct comparison with experiments, particularly regarding small-scale testing of thin films [174,175], micropillars [176,177,178] or nanoindentation experiments [179]. However, the advent of materials informatics has raised new opportunities for the closer coupling of experiments and modeling [180]. The improvements associated with the increase of computational power and the progression in detector technology, with a better acquisition rate and spatial resolution of the detectors used in characterizations, are opening the way to the application of MI techniques in experiments-modeling coupling. Large-scale simulations permit the modeling of more realistic systems and in situ characterization techniques leading to significantly richer data sets that can be used for improving the physical models, the determination of model parameters and the validation of results. A possible application of this coupled approach will be the generation of virtual dislocation microstructures based on in situ experiments to be used as initial conditions for DDD simulations. Although this direct coupling has not been widely investigated so far, several attempts have been made in the generation of synthetic experiments starting from DDD simulations to be used for the application of MI techniques.

## 5. Learning Dislocation Features from Nanomechanics In Situ Experiments

Experimental exploration of optimal metallic alloys with promising mechanical properties at extreme conditions need to be augmented and supported by numerical modeling towards describing physical and chemical mechanisms of plastic deformation. Thus, nanoscale investigations of mechanical deformation (e.g., nanoindentation) are imperative for multiscale modeling and MI strategies for closer coupling between experimental observations and structure-property relationships. However, severe size effects and microstructural noise [82] require statistical tools operating on large data, collected from experiments at (nano-)scales, where comparisons to simulations are viable. For example, validation of numerical simulations can be provided by in situ nanomechanical experiments, thus providing more information about plastic deformation and a detailed characterization of the samples being tested, to enable direct comparison of the time evolution (with mechanical or thermal loading) response of modeling [45,180] This motivates the development of MI software workflows that capture critical resolved shear stress and dislocation behaviors on various slip systems, noting that a detailed analysis of atomic-scale processes of plastic deformation, such as dislocation propagation and twin growth provides an understanding of the defects nucleation, propagation, and multiplication, as well as interactions with other surrounding defects. This aims to create a benchmark of mechanical properties and dislocation dynamics for further experimental data analysis [181] that provides insights into the dislocation reactions at an atomistic level where slip-grain boundary (GB) interactions occur [4,182].

**Figure 9:**
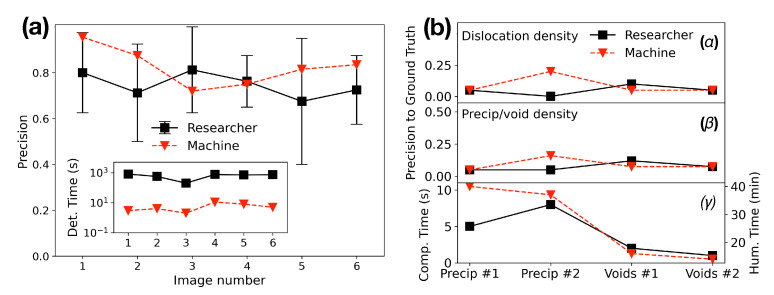
Human Vs. Machine in deformation microstructures. (**a**) A scientific software based on CNN is applied to quantify defects in materials. A comparison of the precision and time efficiency of both the human and machine labeling is presented. The image number corresponds to the selected one from the complete test set and error bars correspond to the minimum and maximum precision obtained by the five researchers. The selected images were also analyzed by five researchers (Redrawn based on results in Ref. [87]). (**b**) A modern electron microscopic system coupled with a CNN based scientific software is applied to directly analyze materials defects. This figure presents the comparison of materials evaluation metrics for defect quantification performed by computer and by expert researchers. Materials metrics include dislocation density in (α), and precipitates and voids number density in (β), the diameter and the time spent for computer and human experts to quantify these defects in (γ) (Redrawn, based on [185]).

A characteristic case of large data, in this context, is the scientific exploration of the detailed characterization of dislocation nucleation and propagation [183], that requires the visualization of dislocations during mechanical loading, by adjusting crystal orientations at every single loading step. The method requires a reconstruction of a large number of 3D frames where materials informatics is involved due to its advantages given by the automated analysis of these emerging and inevitably increasing data generation capabilities to detect and classify materials defects. As an example of MI applications in this context [5,87], a CNN is applied to automate a defect analysis in electron microscopic images. In Figure 9a the authors show the performance of the associated scientific software compared to work performed by five expert researchers (human work) through identifying material defects. Due to observable limitations of human assessment, the authors concluded that the machine performance vs. human performance clearly demonstrated the need for high-quality ML models vs. the ground truth. Into this research scope, modern methods in computer vision and ML have been applied for the statistical representation of microstructure images [184], where reported results show that pre-trained neural networks represented micrographs well with no previous knowledge of the nature of shapes. Likewise, classification in various classes of images has been performed by using deep convolutional neural networks in position averaged convergent beam electron diffraction patterns of scanning 4D scanning-TEM images. Computer vision techniques are certainly very promising for microscopic image analysis by replacing human labor and producing reliable outcomes.

Modern advances in software and the development of fast computer processors potentially increase real-time image recognition, by coupling them with the electron microscopic system to make possible a direct, on-the-fly in situ analysis. Here, efforts have been made to apply CNN architecture to analyze and identify common crystallographic defects in structural alloys like dislocation lines, precipitates and voids [185]. In Figure 9b it is seen that machine vs. human performance, through computing materials’ evaluation metrics for defect quantification, can be comparable for several categories of defect quantification, such as dislocation density, precipitate density, diameter, and diameter standard deviation of precipitates and voids. Overall, automated identification of common crystallographic defects in metals using deep learning semantic segmentation, based on high-quality microscopy data, may provide more accurate results than conventional manual counting, and thus advance model performance.

## 6. Beyond Mechanical Property Empirical Rules: Learning How to Design Metal Alloys

**Figure 10:**
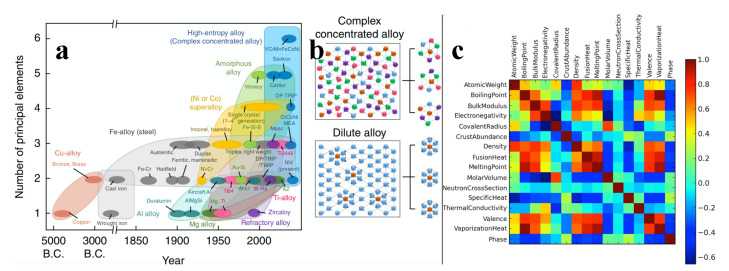
Multi-component solid solution alloys: Solutions for optimal mechanical properties. (**a**) Historical evolution of the compositional complexity of alloys. From simple unary systems to the more complex multi-principal element alloys. (**b**) A comparison of a microstructure in a complex concentrated alloy, in contrast to theoretically tractable dilute alloys (such as steels). (**a**,**b**) Reproduced after [217] based on Creative Commons Attribution 4.0 International License. (**c**) Correlations between various properties in a large collection of HEAs, 1252 observations with 625 single-phase and 627 multi-phase alloys, covering binaries and multi-component systems. Properties such as bulk modulus, valence, vaporization heat, etc., have a relatively strong correlation (<0.6). Reproduced after [218] based on Creative Commons Attribution 4.0 license.

**Figure 11:**
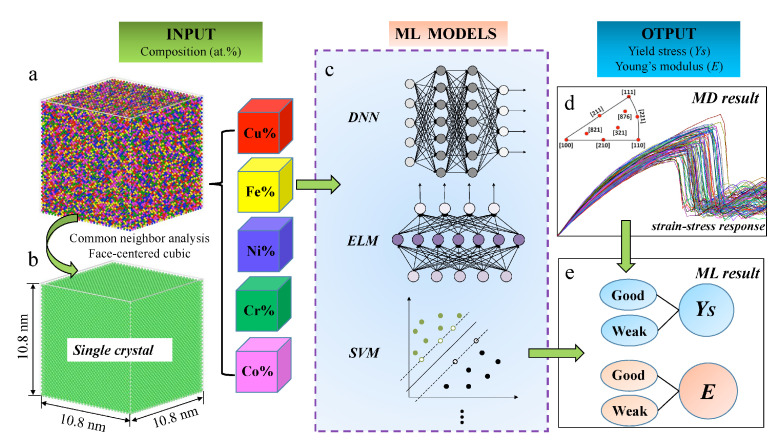
High entropy alloys from multiscale simulations and machine learning. Schematic flow diagram of methods used for tensile tests of 900 HEA single-crystal samples by MD simulation and predicted by 8 ML methods. (**a**) Atomic configuration of a CuFeNiCrCo single-crystal sample, atoms are colored according to the element types. (**b**) Atoms are colored by the common neighbor analysis (CNA) method. The green atoms denote the face-centered cubic structure. (**c**) Schematic of the working principles of some ML models, including deep neural network (DNN), extreme learning machine (ELM), and support vector machine (SVM). (**d**) Stress-strain response of single-crystal HEA samples with various element compositions along [110] orientation by MD simulation. The inverse pole figure indicates nine different crystallographic orientations tested in this study. (**e**) Prediction of the mechanical properties (Ys and E) by ML method. Reproduced after [219] based on Creative Commons Attribution 4.0 license.

While steels drove the industrial revolution, the compositional complexity of modern alloys has dramatically increased during the last century (see Figure 10). Therefore, a necessity for new materials capable to support harsh environments together with the improvement of experimental techniques has made possible the creation of a complex solid solution (CSS) with more than five elements in a single phase [186,187], commonly labeled as high-entropy alloys (HEA). The spatial arrangement of a given phase as well as the particle packing determine the mechanical properties [188,189,190] as well as a large number of electrical and chemical ones of alloys (see, for example, Figure 10 for a correlation analysis). Thus, atomistic simulations can be used efficiently towards the development of MI applications for mechanical property predictions and compositional design (see, for example, Figure 11 for a successful framework for compositional searches and predictions in non-equiatomic HEAs) [191,192,193]. Such MI applications are generally accompanied by post-processing algorithms that allow us to gain some insight into systems and define ML features, by means of assigning a structural type of each particle based on its atomic environment. Among the conventional classification methods, we have the common neighbor parameter (CNP) [194], based on a combination of the common neighbor analysis (CNA) [195,196], and the centrosymmetry parameter (CSP) [197,198]. The idea is to define a parameter as in CSP, but instead of being related to the centrosymmetry of the lattice, the parameter depends on the common atomic neighborhood like the CNA method. Thus, the CNP combines the strength of both CNA and CSP. Therefore their success in correctly describing, for example, amorphous Ni [199], grain boundaries [200,201], metallic glasses [202], deformation of nanocrystalline metals [203,204], and shock deformation of metals [205]. Another frequently used method is the Voronoi analysis [206]. However, due to its high sensitivity to lattice distortions [207,208] the method is generally used to characterize atomic structures of liquid and amorphous systems [209,210,211,212,213,214]. Other less common techniques are also employed to characterize the atomic structures [215,216]. Beyond conventional approaches, MI provides a set of useful tools for the classification of microstructures that emerge in CSSs.

**Figure 12:**
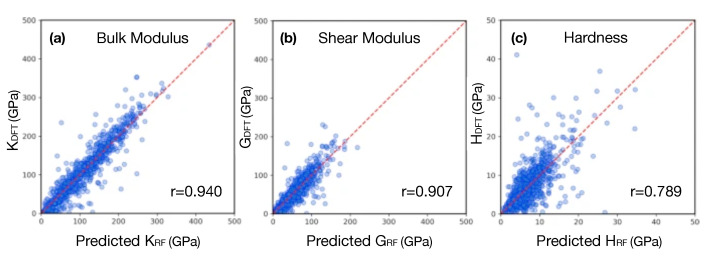
ML and Hardness in multi-component alloys. The Pearson correlation coefficient (r) is utilized as a metric for (**a**) bulk modulus (K), (**b**) shear modulus (G), and (**c**) hardness (H) for 10,421 alloy samples acquired from the Materials Project database [32]. The machine learning models are trained to predict, K, and G, based on density functional theory (DFT) calculations. (Reproduced from [43], based on Creative Commons Attribution 4.0 license.)

ML techniques have proven to be very useful for the discovery of new materials, as well as for the creation of interatomic potentials capable of describing alloys with even more than five elements [220,221]. For this purpose, MI has focused on advances in effectively and efficiently exploring the compositional space to identify novel materials in crystalline and amorphous states. For crystalline solid solutions, the Hume-Rothery rules appear as a first empirical attempt to predict mechanical properties and the solid solution forming ability (SSFA) i.e., the formation of a single crystalline phase, in binary alloys [222,223]. Recently, as a consequence of the discovery of HEAs, the Hume-Rothery empirical rules have been shown insufficient [224,225,226,227]. Replacing these traditional methods with MI approaches has resulted in very accurate predictability of novel high-throughput material design [218,228,229]. Concrete examples of the success of the composition exploring are the material genome project, idealized to uncover properties of inorganic materials [32], Gaussian process classification algorithms towards efficient predictions of promising solutes [230], compositions for optimized mechanical properties (see Figure 12 for an application), the impact of local lattice relaxation to stabilize single-phase of BCC HEA [231], and last but not least, phase prediction of HEAs [43,232,233].

## 7. Materials Deformation Informatics: Challenges, Prospects and Ontology

The key challenge in MI applications for mechanical deformation remains data transferability. Namely, it is imperative to identify concrete ways that the scientific community can re-use scientific data that originate either in experimental or simulation settings [163,180,234,235,236,237], analogous to the exemplary effort being made for DFT data in the Materials Project database [32]. Digitalization is one of the main driving forces of technological and scientific progress and jointly with a variety of quantitative modeling and simulation techniques, digitalization is a passage towards the so-called Industry 4.0 [238], and in particular, it can accelerate the discovery and design of new materials. To predict mechanical properties of materials, many data-based models are incorporated, which is supported by increasingly complex simulations run on increasingly performant infrastructure [239,240]. That leads to the creation of an enormous amount of heterogeneous data, originating from different methods and workflows, maintained by a significant number of groups within different communities.

Managing large data sets is at the core of MI applications, where it allows for obtaining valuable insights on a given material. For example, image-recognition computational techniques rooted in Machine Learning are used in the data analysis of images originating from different experimental sources and various imaging techniques. Machine Learning is also relevant for other data-heavy techniques, where different sets of parameters are considered (data sets with atomic positions, velocities, defects, lengths, orientations, etc.). From the standpoint of data processing, another important domain is 3D visualizations of dislocation nucleation, where materials informatics allows for automated analysis of the emerging defects which inevitably increases data generation capabilities. Data processing and management are very relevant also in the canonical techniques of simulating material properties: Molecular Dynamics (MD) and Monte Carlo (MC). While the MD explores the phase space in a deterministic way by solving the equation of motion for physical evolution, the MC samples the configuration space according to the Maxwell-Boltzmann statistics. Both methods however are very data-intensive and lead to the creation of a very significant amount of data.

The abundance of incompatible data sources often causes reduced interoperability of the data, which in turn prompts the development of model data repositories, often equipped in particular analytical tools, allowing to integrate and share the scattered data [241]. Among the multitude of names, it is worth noting: Integrated Computational Materials Engineering (solids) [242], Computational Molecular Engineering (fluids) [241], Process Data Technology or Computer-Aided Process Engineering (process technology) [243].

The process of digitalization involves two steps: first, data is turned into digital form (digitization), which results in raw unannotated data (dark data) [244,245]. This is then followed by giving the data a suitable structure and description (metadata) to ensure that it remains Findable, Accessible, Inter-operable, and Reusable i.e., conform to the “FAIR" principles [246]. Even though all the FAIR principles are fundamental, mutually dependent, and can not be separated, in some cases (like complex data-driven workflows), it is the interoperability that becomes the most relevant [247]. To provide a shared, standardized representation of the domain knowledge, significant efforts were made towards creating metadata standards and sophisticated classification schemes (ontologies) which are utilized to make complex data standardized, compatible, and efficiently searchable [248].

### 7.1. Interoperability

Interoperability can be loosely defined as an agreement between multiple parties (ideally the whole community) on a common terminology standard, defined by an ontology. The very notion can be split into three main aspects, all originating from theoretical linguistics: syntax, semantics, and pragmatics [238]. Syntactic interoperability is associated with the grammar of a formal language (folders structure, file formats, data items arrangement, etc.), while semantic interoperability is focused on an agreement on the meaning of implications of the data content [249]. Semantic interoperability is only achieved with accepted metadata standards, which support understanding data annotation by all parties through agreed terms. This subsequently allows us to integrate all the communicated data within a single platform with multiple sources and users and ultimately enables cross-system usability [237]. The above two aspects are insufficient without the definition of the context in which the communication occurs and the general understanding of performative roles or, in other words, what different participants in an exchange can reasonably expect from each other [238]. The latter is addressed by pragmatic interoperability, which concerns such requirements as well as recommendations of the practice of communicating and dealing with data [250,251,252].

### 7.2. Metadata

As pointed out, according to the FAIR principles data should be findable, accessible, interoperable, and reusable. Currently, in MI applications for mechanical deformation, experimental data are typically not FAIR, while simulation and ML data approach the FAIR limit. A commonly acknowledged way of conforming the data management to those principles is by incorporating metadata. Metadata can be defined as data about data or more formally, a structured form of knowledge representation [253] which serves the purpose of describing a research asset and its selected aspects. It, therefore, allows to facilitate and improve direct communications of parties exchanging data [238,254] and supports workflows specific to a domain [255]. It is a common practice to divide metadata into categories based on the metadata specificity. Some of them are rather general (for example file sizes, authors, etc.), whereas, others are very particular and only applicable to a single domain. Below, we briefly present the semantic description of four main classes, which are at the core of every sensible data description and hold true for engineering (including modeling materials) and multiple branches of science [238]:

- Technical metadata: Technical aspect of the research asset, mostly the file attributes on a file system level and similar syntactic information (file sizes, checksum information, storage location, access dates, file formats),
- Descriptive metadata: General information about the research asset (authors, keywords, title),
- Process metadata: Information on the generation process of the research asset (for example the computational environment and software used to generate or process the data). It may consist of several consecutive steps,
- Domain-specific metadata: Domain-specific description of the research objects. For example in computational engineering, this includes details about the simulated system, methods of simulation, resolution, etc.

**Figure 13:**
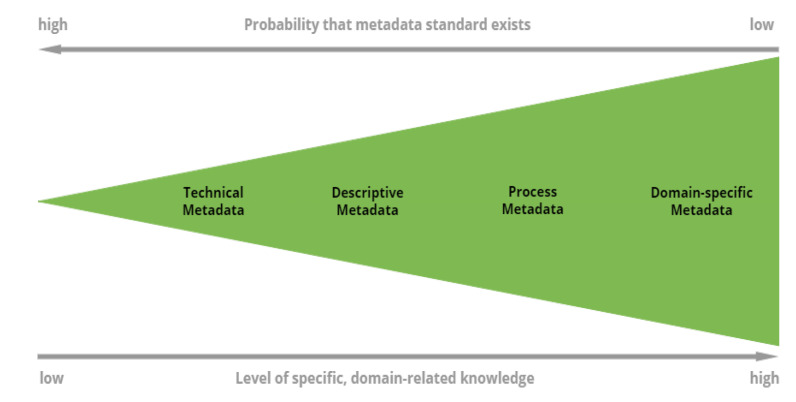
Metadata categories with the specificity level. With an increasing level of specificity, the probability of existing metadata standard is decreasing (Redrawn, based on [238]).

The specificity of the categories is presented in ascending order in Figure 13 [238]. While the technical and descriptive metadata keys are rather generic and relevant for various branches of science, the categories of process metadata and domain-specific metadata are bound to the research process and the research object respectively. To an extent, different classes may overlap and a metadata key can be part of several categories.

The likelihood that suitable standards exist for a given category is smaller for its larger specificity [238] (see Figure 13). While multiple standards exist for technical and descriptive metadata, this does not hold for the process and domain-specific metadata, where a significant development effort is often required. For technical and descriptive metadata, the semantic information is similar throughout all disciplines (DataCite is the standard for a general description and citable data objects [256]). On the other hand, process metadata are deeply connected with the research process, where metadata standards only exist for specific processes (CodeMeta [257], Citation File Format [258]). Similarly for domain-specific metadata, the appropriate standards only exist for few research objects.

Importantly, the distinction between the above four categories is critical also for automated extractability. Technical information is usually easy to extract (mostly file system attributes), and process- and domain-specific information is relatively easy to extract automatically for computational engineering applications. On the other hand, the descriptive information is hardly extractable, since it provides a higher-level description of the research. Therefore, human interpretation is often crucial [238].

### 7.3. Ontologies

**Figure 14:**
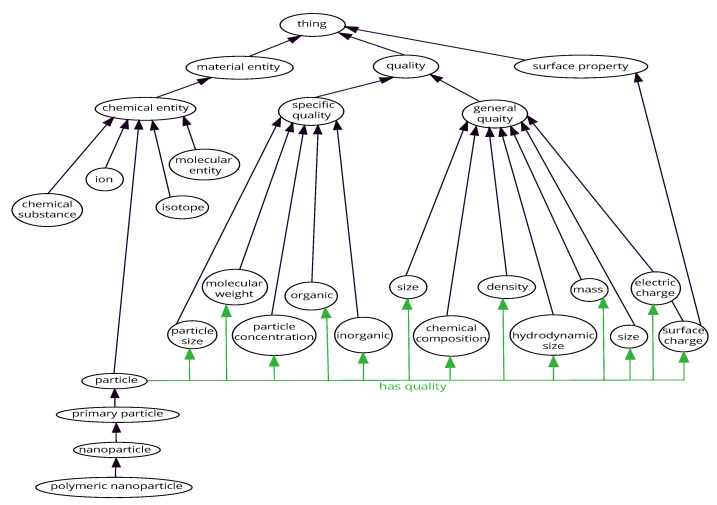
An ontology example: Sample of the NanoParticle ontology (Redrawn, based on [259]).

A convenient way of organizing metadata is by incorporating an ontology, which serves as a framework for metadata design [259]. For mechanical deformation MI applications, such ontologies are currently being pursued. Ontologies and ontology-based techniques are aimed at providing a shared standardized representation of terminology over a domain. In particular, by imposing an ontology-based description of the data, the level of interoperability and reusability is increased, while introducing metadata derived from ontology allows for promoting findability and accessibility. Ontologies are defined as sophisticated classification schemes aimed at making complex data searchable. They have a hierarchical structure and define the basic terms and relations over a domain, as well as the rules for combining them [259]. Ontologies consist of four components: (i) concepts that represent sets or classes of entities in a domain, (ii) instances that represent the actual entities, (iii) relations (is-a or has-a), and (iv) axioms representing restrictions imposed on the domain. The tree-like ontology structure allows organizing entities based on their granularity, from general to specific. The actual set of components might be the basis for ontology classification; for example, in Figure 14 a little sample of NanoParticle ontology [260] is presented, where the black arrows represent axioms of is-a relations, i.e., if A is B, then all entities belonging to A belong to B as well. A is referred to as a sub-concept of B. In this example, a chemical substance, particle, ion, isotope, and molecular entity are all sub-concepts of a chemical entity. Therefore, all chemical substances, particles, ions, isotopes, and molecular entities are also chemical entities. Furthermore, all primary particles are particles, all nanoparticles are primary particles, and so on. It is clear that the is-a relation is transitive i.e., describes inheritance of properties. Another important relationship is the has-a relation, which is associated with the axiom that entities have qualities (green arrows).

Metadata modeling can be viewed as an intermediate component between non-formal data descriptions and complete formalizations of metadata keys [238,261]. Its purpose is to describe a research object as well as its relation to other objects, which derives from the common understanding of the domain. This approach could also be called ontology-based metadata, given that the metadata model is build based on an object model [238].

The process of engineering a hierarchical metadata model includes several consecutive steps, some of which might be repeated until a detailed enough description of the research is reached. In the first part, a clear understanding of the research object is reached, which is achieved by the analysis of the research process with all potentially involved stakeholders and using a natural language. In this step, the information about relevant entities is found and grouped, which is subsequently followed by identifying the attributes describing entities (names, units, etc.) as well as terms, relations, and rules (in material modeling an example of relevant entity is a component, which represents a chemical species). Everyone potentially involved contributes to this step, since the metadata will serve as a semantic convention for further communication. Finally, the relations between the entities must be understood, including how different entities are connected to create an in-depth description. This part is subjective, arbitrary, and strongly specific to the research [238].

Once the object model is defined in a formal language, some attention has to be paid if its certain parts exist in other standards (the more general metadata, the more probably they are to be found in other metadata standards). In the last step, the metadata model representation is created in terms of choosing the formal language. In most situations, it is XSD or JSON, which offers the possibility of defining the entities, attributes, and relations.

## 8. Conclusions

In this review, we explored core MI applications on mechanical deformation properties, using various approaches, and various ML methods and frameworks, especially relevant to a broad range of elasticity, plasticity and failure properties, as well as composition dependences, with particular focus on concentrated solid solution alloys. Starting from what an experimenter can see, in testing and microscopy experiments, MI and ML frameworks help quantify defect and phase densities that may control yielding or/and damage properties. However, greater progress in MI for mechanical deformation and composition dependencies requires the understanding of physical mechanisms at the nanoscale, with consequent demand for defect characterization through MI methods, such as dislocation ensemble classification.

The MI capacity has been highlighted in various contexts related to microstructural defect classification, in situ characterization, constitutive modeling, property prediction, and materials discovery and high-throughput design, in both supervised and unsupervised settings, with multi-scale and multi-dimensional (simulation-based and/or experimentally-derived) datasets that essentially unveiled novel structure-property relationships. However, a key challenge in data-driven research of materials is the smooth and reproducible access to well-maintained and reusable databases which are diverse and heterogeneous in nature, as noted earlier in Section 7. Sources of disparities in data are typically diverse and often stem from different measurement techniques and associated uncertainties, various experimental conditions, and underlying assumptions. Therefore, it is quite likely that a careless combination of information from different data repositories often leads to misinterpretation of spurious trends and significant bias in predicted properties. This, in fact, highlights a need for human supervision in building meaningful synergies among diverse data sources to ensure the soundness and robustness of the machine-generated output.

In conclusion, despite MI successes, it is clear that the future appears bright and may hold many positive surprises. The key step for MI applications to achieve the ultimate goal, is to consistently accomplish feats that human researchers have not suspected or predicted. In the realm of multi-component concentrated alloys, such an ultimate goal may be defined as the clear, reproducible prediction of novel alloys that display exceptional physical and especially, mechanical properties, even in extreme conditions. While there is still a lot of proving ground for MI to navigate, the reviewed MI trials have shown a consistent and robust trend: Using analogies, similarities, and pattern constructions and classifications, MI emerges as the reliable and helpful companion of any materials scientist.
